# A unique and colossal development in global health

**DOI:** 10.5588/pha.23.0049

**Published:** 2023-12-07

**Authors:** G. N. Kazi

**Affiliations:** Dopasi Foundation, Islamabad, Pakistan

Professor Mario C. Raviglione possesses a rare blend of qualities. Having worked in highly pivotal positions at the WHO for over 25 years, he is one of the foremost health academicians in Europe. In addition, he is one of the few pioneers of TB control who are not ‘territorial’ – indeed he encourages the need to adopt a more holistic approach to ending TB as it is beyond the health sector capacity of a country.^1^–^4^ It is not possible to sum up all his achievements in a short note, but let me just say that with this book he has triumphed yet again. Together with associates, Professor Raviglione has edited a highly comprehensive and self-contained book on global health entitled ‘Global Health Essentials’,^5^ which is based on WHO guidelines. The book runs to nearly 600 pages, and in his foreword, WHO’s Director General Dr Ghebreyesus notes that the work highlights the need to ‘rethink global health in light of the lessons learned and challenges resulting from the COVID-19 pandemic and other emerging global health threats, including environmental and climate changes’. These affect the health of the planet as a whole and he asserts that the book promotes global solidarity and social justice, which is critical to positively impacting people’s health.

Global Health is a multi-dimensional discipline, so the breadth of the book is awe-inspiring. After introducing Global Health, the authors discuss the global burden of disease across all sub-sectors: health through the life course, including maternal and child care; mental health and geriatric care; communicable and non-communicable disease control; social determinants of health; health systems, including governance and global financing issues; innovations in global health such as genomics, digital technologies and the continuum of research; linkages among the Sustainable Development Goals, global health laws, public-private mix in health, impact of climate change; and methods in global healthcare, including epidemiology, biostatistics, disease modelling, surveillance, monitoring, evaluation, and priority setting, and achieving cost-efficiency through a global health project management for the entire planet. One Health approaches are also discussed at length in a user-friendly manner. Within the realm of health research, applied research, translational research, operational research and policy and systems research have been identified as the key areas. Inter-disciplinary research bringing about synergy among different sectors, researchers, ­donors, research institutions, programmes, health systems, patients and advocacy groups has also been supported ostensibly to facilitate adequate budgetary allocations for the purpose. Dr Raviglione has himself written the first chapter on the definition, principles and evolution of global health. This aims to put an end to any unnecessary controversies and confusion related to the terms ‘global health’ and ‘public health,’ creating an environment that is conducive to following the defined path and addressing sensitive topics as we progress. A chapter on global health diplomacy will help to soothe any frayed nerves. From the perspective of low-income countries, it was exciting to read the chapter on financing common global goods for health and the means to bridge the financial gaps. Interestingly, the book discusses the international politics surrounding COVID-19, while stressing the need for equity, inclusiveness and diversity as the critical drivers of global health. Equity forms the basis and foundation of the primary healthcare (PHC) philosophy, in line with WHO’s Constitution, and permeates throughout this succinct, but comprehensive work. The PHC approach, which involves decentralisation may help to attain universal healthcare and other critical elements of SDG-3 relating to health, as highlighted by several recent articles in *Public Health Action*.^6^–^8^ It is significant that this book was launched at a time when the United Nations General Assembly was holding high-level meetings on global pandemic prevention, preparedness and response, universal health coverage and the elimination of tuberculosis.^9^

The book is not only a must read for students of global health but also for all physicians, other health professionals, policymakers, planners, social sector economists and decision makers. This applies at all levels globally, regardless of the socio-economic challenges and health issues within a specific country. I wholeheartedly congratulate Professor Raviglione, his fellow editors, and all those who have contributed to this monumental effort.
